# Social and Individual Subjective Wellbeing and Capabilities in Chile

**DOI:** 10.3389/fpsyg.2020.628785

**Published:** 2021-01-18

**Authors:** Pablo A. González, Francisca Dussaillant, Esteban Calvo

**Affiliations:** ^1^Department of Industrial Engineering, Faculty of Physical and Mathematical Sciences, Centre for Public Systems and Center for Research in Inclusive Education, Universidad de Chile, Santiago, Chile; ^2^London School of Economics and Political Sciences, International Inequalities Institute, London, United Kingdom; ^3^Ministry of Finance, Santiago, Chile; ^4^Society and Health Research Center, School of Public Health, Universidad Mayor, Santiago, Chile; ^5^Laboratory on Aging and Social Epidemiology, Facultad de Humanidades, Universidad Mayor, Santiago, Chile; ^6^Department of Epidemiology, Mailman School of Public Health, Columbia University, New York City, NY, United States; ^7^Robert N. Butler Columbia Aging Center, Mailman School of Public Health, Columbia University, New York City, NY, United States

**Keywords:** social belongingness, quality of life, subjective wellbeing, capabilities, social wellbeing

## Abstract

The notion of social belongingness has been applied to different scales, from individual to social processes, and from subjective to objective dimensions. This article seeks to contribute to this multidimensional perspective on belongingness by drawing from the capabilities and subjective wellbeing perspectives. The specific aim is to analyze the relationships between capabilities—including those related to social belongingness—and individual and social subjective wellbeing. The hypotheses are: (H1–H2) There is a relationship between capabilities (measured as evaluation and functioning) and (H1) individual and (H2) social subjective wellbeing; (H3) The set of capabilities associated to individual subjective wellbeing differs from the set correlated to social subjective wellbeing; (H4) The intensity and significance of the correlation between subjective wellbeing and capabilities depends on whether the latter is measured as evaluation or functioning; and (H5) The relationships between capabilities and subjective wellbeing are complex and non-linear. Using a nationally representative survey in Chile, multiple linear (H1–H5) and dose response matching (H1–H5) regressions between capabilities and subjective wellbeing outcomes are estimated, confirming all hypotheses. Subjective evaluations and effective functionings of some capabilities (“basic needs,” “social ties,” “feeling recognized and respected;” “having and deploying a life project”) are consistently correlated with both subjective wellbeing outcomes. Others capabilities are correlated with both subjective wellbeing outcomes only when measured as functionings (contact with nature), do not display a systematic pattern of correlation (“health,” “pleasure,” “participation,” and “human security”) or are not associated with subjective wellbeing (“self-knowledge” and “understanding the world”). When observed, correlations are sizable, non-linear, and consistent across estimation methods. Moreover, capabilities related to social belongingness such as “social ties” and “feeling recognized and respected” are important by themselves but also are positively correlated to both social and individual subjective wellbeing. These findings underscore the need of a multidimensional perspective on the relationships between capabilities and subjective wellbeing, considering both subjective and objective, as well as individual and social aspects that are relevant to belongingness. These findings also have practical and policy implications, and may inform public deliberation processes and policy decisions to develop capabilities, promote subjective wellbeing, and ultimately promote positive belongingness.

## Introduction

The notion of social belongingness has been applied to different scales, from individual to social processes, and from subjective to objective dimensions. This article contributes to this multidimensional perspective on belongingness by drawing from the capability approach (human development) and subjective wellbeing perspectives.

Subjective wellbeing is traditionally defined as a subjective evaluation of our own life, an hedonic balance of feelings, emotions, and appraisals that can be adequately assessed by self-reported questionnaires (Diener et al., 2018; Das et al., 2020; Karunamuni et al., 2020). This concept of individual subjective wellbeing is often equated to happiness and life satisfaction (Veenhoven, 2018; Bergsma et al., 2020).

Subjective perceptions and evaluations of wellbeing can explicitly refer to our experience and evaluation of society and not only our inner world. Social wellbeing is a multidimensional and complex concept that admits several possible definitions. Keyes (1998) suggests it is composed of five dimensions: social integration; social contribution; social coherence; social actualization; and social acceptance—that require different scales for its measurement. A close concept is social capital (Coleman, 1990), which has been related to health outcomes (Choi et al., 2014; Ehsana et al., 2019). So is social cohesion, which is constituted of several dimensions such as: social integration, identification or sense of belongingness; orientation toward the common good; shared values; degree of inequality between individuals and groups within society; society’s ability to secure the long-term wellbeing of its members (Oyanedel and Páez, 2020, in this special issue). Social wellbeing and social cohesion are similar except that the former has a meaning at the individual level aside the collective group. It is a measure of how each individual experience society rather than a characteristic of a social collective. Non -liberal political philosophy approaches such as communitarian, republican and social communication perspectives might provide solid philosophical foundations for these concepts and why we should care about them (Habermas, 1998).

The capability approach provides a framework for a more complete assessment of quality of life that arises from Sen (2009) critique to subjective wellbeing and Aristotle’s concept of a good life (Nussbaum, 2000). The capability approach proposes to assess quality of life according to three concepts: capabilities, defined as the freedoms of doing and being that people have reason to value; functionings, which are actually achieved states of being and doing; and agency, the capacity of the individual to pursue his/her own objectives^1^.

Drawing on a multidimensional perspective integrating these concepts, the objective of this study is to analyze the relationships between capabilities with social and individual subjective wellbeing outcomes using a representative sample of Chilean adults.

The hypotheses are: (H1) There is a relationship between capabilities (measured as evaluation and functioning) and individual subjective wellbeing; (H2) There is a relationship between capabilities (measured as evaluation and functioning) and social wellbeing; (H3) The set of capabilities associated to individual subjective wellbeing differs from the set correlated to social subjective wellbeing; (H4) The intensity and significance of the association between subjective wellbeing and capabilities depends on whether the latter is measured as evaluation or functioning; (H5) The relationships between capabilities and subjective wellbeing are complex and non-linear.

The paper is organized as follows. Section 2 discusses the measurement of subjective wellbeing and capabilities. Section 3 revises the literature on the relationship of subjective wellbeing and capabilities. Section 4 briefly presents the data and methodology. Section 5 turns to the results using two statistical methods: linear regression and dose-response matching. Section 6 discuss limitations, implications and challenges ahead. Section 7 concludes.

## Measurement of Subjective Wellbeing and Capabilities

Measurement of individual subjective wellbeing or happiness has made significant progress in recent years. Several indicators are actually used in different surveys at a national and international level (such as the World Values Survey and the Gallup Poll) and the World Happiness Report updates every year about its evolution in about 156 countries and 186 cities. Of the plethora of indicators proposed in the literature that involve hedonic, psychological and evaluative approaches (OECD, 2013), it is the latter that is more coherent with the capability approach (Anand et al., 2009) and is the one preferred in the present study.

In contrast, there is no consensus on how social wellbeing (or its opposite, social discontent) should be measured. Keyes (1998) provides a sound multidimensional scale that has been validated, but is rather long to apply. Many studies use other measures, or concentrate on particular dimensions, such as social belongingness or trust, as reviewed by Oyanedel and Paez in this special issue. Social subjective wellbeing refers to how individuals experience and evaluate their society. It is composed of their own experience with others as well as their experience on how others experience society. It is not possible to address it directly through one direct question as it is composed of several latent dimensions. For the present study a composite index of trust in institutions and evaluation of social opportunities is used.

On the other hand, the measurement of capabilities is at its early stages of development. There are several attempts to measure capability indicators based on questions in existing surveys (for instance Anand et al., 2005; Ramos and Silber, 2005; Veenhoven, 2010; Muffels and Heady, 2013; Graham and Nikolova, 2015), following Anand and van Hees (2006) suggestion that survey questions about the “scope to achieve things” and “limitation of opportunities” can capture capabilities. There are also a few specially designed questionnaires (Anand and van Hees, 2006; Anand et al., 2009, 2011; Simon et al., 2013).

Anand et al. (2009) seminal contribution operationalized Nussbaum (2002) list of capabilities with 65 questions in a survey applied to 778 individuals representing the population of England, Scotland and Wales. This list of capabilities was compared to the set contained in the British Household Panel Survey, which was evaluated as incomplete in relation to the list proposed by Nussbaum, henceforth the need to take special surveys designed for this purpose.

The capability approach has also been adapted to assess the health situation of older people. Makai et al. (2013) developed the ICECAP-O with ICE referring to Investigating Choice Experiments and CAP-O referring to a CAPabilities measure for Older people and Al-Janabi et al. (2012, 2013) developed ICECAP-A for Adults. For the measure for adults, the five dimensions are stability, attachment, achievement, autonomy and enjoyment. The five dimensions used in ICECAP-A reflect an interpretation of the capabilities framework that is inspired by the healthcare background of the research. Van Ootegem and Verhofstadt (2015) propose the question “How do you consider your possibilities/opportunities in life in general?” which refers to opportunities and therefore is more forward looking as compared to the more backward looking life satisfaction, as an aggregate measure of capabilities.

## Relationship Between Subjective Wellbeing and Capabilities

When it is recognized that wellbeing is multidimensional, the issues of how to measure an individual’s wellbeing, compare different individuals or assess a given situation becomes more complicated, as it requires weighting heterogeneous dimensions. Sen (2009) suggests this weighting should be part of the deliberation process whereby each society defines what capabilities should be valued. Although it is clear that both Sen and Nussbaum downplay the role of subjective wellbeing in their proposals for assessment of both wellbeing and social justice—at the most as one functioning among many—a few studies have attempted to link capabilities and subjective wellbeing (see Comim, 2005, and the references summarized in this section). An obvious link is to use the relationship between different capabilities and subjective wellbeing as a first approximation to weighting capabilities as suggested by Binder (2014). Capabilities might be explanatory variables of individual subjective wellbeing and the resulting estimated coefficients might be a proxy for the weight that should be given to each capability. Similarly, Binder (2014) suggests public policy should concentrate on those capabilities that are relevant to subjective wellbeing^2^ Nevertheless, as suggested by Frey and Stutzer (2010), these technical exercises might be interesting to consider in democratic deliberation but should never replace this process, as elected representatives, not experts, should take and be responsible for political decisions. This is the perspective followed by this article, as its objective is to determine what capabilities are more correlated to subjective wellbeing, considering not only individual subjective wellbeing as suggested by Comim (2005) and Binder (2014), but also social subjective wellbeing.

In fact, previous empirical research has established a correlation between certain capabilities and individual subjective wellbeing (Anand et al., 2005, 2009; Anand and van Hees, 2006; Van Ootegem and Spillemaeckers, 2010; Veenhoven, 2010; Muffels and Heady, 2013; Graham and Nikolova, 2015; Yeung and Breheny, 2016). Starting with those studies using their own measure of capabilities, Anand et al. (2009) found that only 17 of their 65 (dimensions of) capabilities were correlated at a 95% confidence level with life satisfaction (LS), which is their preferred indicator of subjective wellbeing on the grounds of its coherence with Sen-Nussbaum capability approach. The list is further reduced when other controls are included and only three are significant for all age groups: love-care-support; life project; and usefulness/inclusion^3^. “Adequacy of accommodation”^4^ matters only for those below 45 years old while “capacity to express feelings”^5^ is important for the older group.

Yeung and Breheny (2016) used Structural Equation Modeling to estimate the impact on Subjective wellbeing measured as life satisfaction, happiness and quality of life of: (i) commodities (measured as total number of chronic illnesses reported, and physical and mental health scores); (ii) personal and environmental factors (economic living standard and everyday discrimination) and (iii) capabilities measured by the LSCAPE (Living Standards Capability for Elders) that assess the extent to which older people are capable of achieving valued functionings across six domains: health care, social integration, contribution, enjoyment, security and restriction. Functionings were assessed in terms of activity participation as this measures extent of participant achieved. Wellbeing comprised three single items of life satisfaction, happiness and quality of life.

A different exercise is performed by Van Ootegem and Verhofstadt (2015), who explored the relationship of an aggregate capabilities indicator with the realizations for nine life domains (related to those mentioned in Stiglitz et al., 2009)^6^, standard socioeconomic controls and personality traits using a sample of 2,990 respondents representative of the Flemish population of Belgium. A similar exercise was performed for life satisfaction. All life domains are significant for the aggregate indicator of capabilities while only education is not for life satisfaction.

There are other studies that use existing data sets that contain information related to the capability approach instead of a specially designed survey to measure capabilities. The selection of capability proxies based on existing data is subject to epistemological errors and constrained by data availability that might leave important capabilities unmeasured (Graham and Nikolova, 2015). Muffels and Heady (2013) examined the impact of capabilities on life satisfaction (as well as relative income and employment security) using random and fixed effects GLS models in 25 years of German and 18 years of British panel data. Capabilities are interpreted in terms of the amount of four types of capital: economic (wealth, human capital endowments and skills); social (level of trust in other people and the social networks people are involved in, indicated by the frequency of contact with others and the support people get from others in their network, but also the membership of organizations and associations or clubs such as trade unions, social and sport clubs); cultural (individual values and life goals, such as work, family and social values like helping others and volunteering, and life goals such as forming a family, raising children or making a career and to risk attitudes such as risk taking or risk aversion); and psychological (people’s personality traits), This choice is probably attributable to data limitations of existing data sets. While interpreting some of these assets as capabilities instead of standard socio-economic and personality controls might be open to controversy, Muffels and Heady (2013) found that “capabilities” pertaining to human capital, trust, altruism and risk taking, and choices to family, work-leisure, lifestyle and social behavior strongly affect long-term changes in subjective and objective wellbeing though in a different way largely dependent on the type of wellbeing measure used.

Graham and Nikolova (2015) uncovered a positive relationship between capabilities and different measures of individual subjective wellbeing (best possible life, experienced happiness yesterday, experienced stress yesterday, experienced anger yesterday) using the Gallup World Poll. Two types of measures of capabilities are distinguished. On the one hand, perceptions of capabilities and means, which include: no health problems; belief in hard work for getting ahead; and satisfied with freedom in life. On the other hand, objective capabilities and means include: some college/college diploma; household income; and employment categories. As in the former study, some of the variables might not be adequate measures of capabilities while other capabilities are clearly not included.

It is interesting to note that Graham and Nikolova (2015) explored the effect of different levels of capabilities by using quantile regression. This method allowed distinguishing that while education is positive for subjective wellbeing in general, it is negatively correlated with subjective wellbeing at the top of the happiness distribution. The authors hypothesized this could be due either to the fact that learning makes the “happy peasants” realize their absolute or relative deprivation and lack of choice and opportunities or that the most educated have unrealistic expectations and ambitions and even their autonomy and capabilities cannot make them happy. It is likely, therefore, that the same capabilities and means have different meaning and importance for people at different points of the subjective wellbeing distribution.

Suppa (2015) approximated capability deprivation through the combination of “inadequate income” together with non-consumption data of “pivotal goods” using German panel data. This proxy for capability deprivation reduces life satisfaction significantly and individuals fail to adapt within the subsequent 4–6 years.

Finally, Steckermeier (2020), using multiple linear regressions, estimates a positive effect of six basic functionings (safety, friendship, health, financial security, leisure, and respect) on people’s life satisfaction in 33 countries covered by the 2016 European Quality of Life Survey. The positive “effects” of some capabilities is reduced when people experience a great deal of autonomy over their lives or when societal conditions provide people with more opportunity and choice.

On a more theoretical level, Veenhoven (2010) and Pugno (2015) suggest that both capabilities create happiness and happiness enhance capabilities. Pugno (2015) suggests a two-way causation: from functionings and underlying conditions to wellbeing and an increase in personal conversion factors; and from wellbeing, when personal conversion factors increase, to better and new functionings. This finds some empirical support in Anand et al. (2011)^7^ and Binder and Coad (2011)^8^.

Contrary to a burgeoning literature on the relationship between capabilities (or more precisely realized capabilities or functionings) and individual subjective wellbeing mostly measured as life satisfaction, there is little study of the relationship of capabilities and social wellbeing. The most related to the issue is Lanzi (2011), who analyzed the relationship between the capability approach and social cohesion, from a revision of socio-psychological literature on cohesiveness in groups and communities. It is suggested that this lack of research on the effects of capabilities on social cohesion (and vice versa) is due to the fact that Sen’s perspective on wellbeing is ethically and methodologically individualistic (Gasper, 2002). Therefore, Lanzi (2011) proposed to use Ibrahim (2006) concept of social capabilities that individuals might attain by virtue of their engagement in collective action or their membership of a social network. Instead of considering the effect of capabilities on cohesiveness, he examined the relationship in the other direction, suggesting that social cohesion has positive effects on the development of social capabilities and human wellbeing. However, he also suggested cases and conditions in which stronger social cohesion might delay the achievements of certain excluded and dominated groups.

## Data and Methods

### Sample

This study uses a special designed survey of capabilities and subjective wellbeing. The sample consists of 2.535 cases and is representative of the urban and rural Chilean population aged 18 years old and above (12.584.252 people). Data was gathered on a face-to-face mode, at the respondent’s home, between July and September 2011 in the framework of UNDP national Human Development Report. Maximum sampling error was 1.9% with a 95% confidence level and estimated effect of design of 1.12.

Details of the three-stage stratified by conglomerate sample design; interview questions and further statistics can be found in the appendix to González et al. (2012).

### Measurements and Instruments

Starting from the basis of the capability lists that have been proposed and used by Alkire (2002) and Nussbaum (2002), Sen’s (2009) suggestion on deliberation was followed, although restricted to small groups from different socioeconomic sectors. This led to minor changes in the list that were operationalized with questions that referred to subjective evaluation of capability and functionings. The list included health; basic needs (housing and income); self-knowledge; understanding the world (education); experience pleasure and emotions; being in contact with nature; participation and influence; social ties (friends, partner, family); feeling recognized and respected in dignity and rights; having and deploying a life project; human security (freedom of fear). The operationalizing questions are presented in Table 1.

**TABLE 1:** Capability indicators.

	**Subjective evaluation**	**Effective functioning**
Health	Overall, in balance, your health is: very good (11%), good (42%), average (38%), bad (7%), very bad (2%)	In the last 12 months, did you experience any physical health problem that has limited your daily activities more than ten consecutive days (Yes 23%/no)
Income	Thinking on the total income of your family, would you say that…?: It is not sufficient, you experience great difficulties (7%); it is not sufficient, you experience difficulties (27%); it is just adequate (51%); it is more than enough, you can save (15%)	In which bracket is the monthly income of your family? Less than US$300 (14%); 300–450 (19%); 450–600 (13%); 600–750 (8%); 750–920 (8%); 920–1,150 (6%); 1,150–1,460 (6%); 1,460–1,970 (3%); 1,970–3,000 (4%); 3,000–6,000 (4%); more than 6,000 (2%); don’t know (12%)
Housing	Questions related to the perception of the material quality of the house, its basic services, its appearance, and its space	Availability of drinking water, sewerage and hot water
Inner life and self-knowledge	I am a person with a rich inner life. Strongly agree (26%); agree (58%); disagree (12%); strongly disagree (2%) (no answer 2%).	With what frequency would you say that you think about things that happen to you and take time to think about yourself? Very frequently (31%); with some frequency (40%); with small frequency (23%); with very small frequency (6%).
	I am a person that knows him/her self very well. Strongly agree (27%); agree (59%); disagree (11%); strongly disagree (1%) (no answer 2%).	
Understanding the world we live in	Different events, both in Chile and abroad, can affect your life. How well informed do you feel about those facts… Very informed (21%); Informed enough (44%); Little informed (31%); Uninformed (4%).	What is the highest level of education you completed? (if studying, what is your current grade) Incomplete basic (1–8) (16%); graduate basic (13%); incomplete secondary (14%); complete secondary (27%); incomplete vocational higher education (5%); complete vocational higher education (6%); incomplete university (8%); complete university (9%); postgraduate studies (1%)
Experience pleasure and emotions	(The respondent has been previously asked about the accomplishment of different activities) Thinking about the activities that you enjoy the most, would you say that… You carry them out as much as you like (9%); almost as much as you like (33%); less than what you would like (38%); much less than what you would like (19%).	Frequency of recreational activities: read a book; listening to music; taking a nap; dancing; practicing a hobby; going out to the cinema or theater; concerts; stadium Practicing sports
Enjoying and feeling part of nature	Would you say that you go to parks and green areas: As much as you like (11%); almost as much as you like (22%); less than what you would like (37%); much less than what you would like (29%).	In the last month, with what frequency did you go to parks and green areas? Every day (4%); Many days a week (8%); Once a week (11%); 2–3 times a month (14%); once a month (19%); never (42%).
	Would you say that you do activities in contact with nature: As much as you like (12%); almost as much as you like (25%); less than what you would like (37%); much less than what you would like (25%).	In the last year, with what frequency did you do activities in contact with nature? More than 6 times (19%); 4–5 times (8%); 2–3 times (21%); once (21%); never (30%).
Participation and influence in society	How much do you agree with the following sentence: “People like me can do a lot to solve the problems that affect their neighborhood or community”: Strongly agree (11%); agree (47%); disagree (31%); strongly disagree (9%).	Do you participate actively in an organization such as sports club, religious group, neighborhood organization, trade union, cultural group or other? (Yes 32%/No 68%)
	How much do you agree with the following sentence: “People like me can do a lot to change the course of the country”: Strongly agree (14%); agree (47%); disagree (29%); strongly disagree (8%).	In the last 3 years, did you have an active participation in: Public manifestations (9%); Taking a letter, claim or request to some authority, company or media (10%); Create or support an internet campaign (10%).
		During the last 3 years, did you have an active participation in a solidarity campaign or volunteer work (17%).
Social ties	How much do you agree with the following sentence: “I feel I am a loved and valued person”: Strongly agree (40%); agree (52%); disagree (6%); strongly disagree (1%).	Frequency of carrying out of the following activities: sharing with friends; going out with (girl/boy friend-wife/husband); sharing with own children
	How much do you agree with the following sentence: “I frequently feel lonely”: Strongly agree (8%); agree (25%); disagree (48%); strongly disagree (19%).	With how much frequency do you do the following activities with your family? Talk about family matters: Usually (52%); with some frequency (25%); only in a few occasions (18%); never (6%).
		With how much frequency do you do the following activities with your family? Going out together: Usually (41%); with some frequency (22%); only in a few occasions (25%); never 11%).
		During the last month, how often have you been invited by friends to go out or to their home: More than once a week (19%); 2–3 times a month (32%); once (18%); never (31%).
	How much do you agree with the following sentence: “People around me care a lot about me”: Strongly agree (38%); agree (50%); disagree (8%); strongly disagree (2%).	With respect to friendship, would you say that…: You have lots of friends (21%); you have few friends (54%); you do not have friends, only acquaintances (24%).
Feeling recognized and respected in dignity and rights	How much do you agree with the following sentence: “I feel that in this society the dignity and rights of persons like me are fully respected”: Strongly agree (5%); agree (36%); disagree (43%); strongly disagree (15%).	In general terms, how often would you say that you experience situations of maltreatment: with very small frequency (42%); with small frequency (40%); with some frequency (14%); very frequently (3%).
		In general terms, how often would you say that you experience situations of discrimination: with very small frequency (44%); with small frequency (38%); with some frequency (14%); very frequently (4%).
Having and deploying a life project	In relation to your personal project and goals, would you say that: you are doing: nothing to achieve them (4%); less than what is necessary to achieve them (17%); almost all what is necessary to achieve them (44%); everything necessary to achieve them (35%).	How defined would you say your life project is? Very defined (33%); some definition (36%); little defined (18%); not defined (14%).
Feeling secure and free from threats	Think about needing to get medical attention in case of a catastrophic or chronic illness such as cancer or heart attack. How confident are you on: receiving a timely attention; being able to pay for the costs not covered by your health insurance; the quality of the service received. Total confidence (11; 7; 10); enough confidence (31; 22; 32); some confidence (41; 38; 32); no confidence at all (16; 31; 18).	Do you have health insurance? Yes (92%)
	If today you wanted to find an acceptable remunerated job, how easy would it be for you? Very easy (4%); easy (21%); difficult (41%); very difficult (32%).	In your current job, do you have a written contract? Yes (63%)
	Thinking about your current job, how much confidence do you have in not losing it in the next 12 months? No confidence at all (8%); little confidence (19%); enough confidence (36%); total confidence (36%).	
	If you lose or leave your actual job, how difficult would it be for you to find an acceptable new job? Very difficult (17%); difficult (41%); easy (32%); very easy (7%).	

*Own elaboration based on González et al. (2012).*

Individual subjective wellbeing was measured by the question: “Overall, how satisfied are you with your life as a whole at this moment?” The answer is a scale from 1 to 10, where 1 means “completely unsatisfied” and 10 means “completely satisfied.” On the other hand, social subjective wellbeing was approximated through a composite index of trust in institutions (Catholic church, Evangelic church, Media, Courts, Government, Political parties, Congress, Corporate sector, Municipality, social and citizen organizations) and evaluation of opportunities offered by the country to its people (good health, human security, satisfy basic needs, participation in decision making, been educated and well informed, freedom to decide what people want to do with their life). All questions were considered in its positive sense to allow for aggregation. Table 2 shows the descriptive statistics of the resulting indexes.

**TABLE 2:** Descriptive statistics.

	**Mean**	**Standard dev.**	**Min.**	**Max.**
**Wellbeing indexes**				
Life satisfaction	7.27	2.14	1	10
Social subjective wellbeing	4.83	1.69	0	10
**Capabilities as functionings**				
Health	0.77	0.42	0	1
Participation	0.39	0.25	0	1
Security	0.71	0.22	0	1
Understanding	0.49	0.21	0	0.96
Nature	0.34	0.29	0	1
Self-knowledge	0.65	0.29	0	1
Needs	0.61	0.20	0	1
Pleasure	0.76	0.21	0	1
Respect	0.84	0.21	0	1
Ties	0.60	0.21	0	1
Project	0.63	0.34	0	1
**Capabilities as subjective evaluation**				
Health	0.63	0.21	0	1
Participation	0.55	0.22	0	1
Security	0.38	0.16	0	0.92
Understanding	0.60	0.27	0	1
Nature	0.40	0.28	0	1
Self-knowledge	0.71	0.18	0	1
Needs	0.63	0.20	0	1
Pleasure	0.44	0.30	0	1
Respect	0.44	0.26	0	1
Ties	0.70	0.19	0	1
Project	0.66	0.27	0	1

*Own elaboration based on González et al. (2012).*

### Analytical Techniques

Two estimation methods are presented. First, multiple linear regression models were used to estimate the relationship between the two subjective wellbeing outcomes and all 11 capabilities simultaneously. Two models were estimated for each outcome (life satisfaction and social subjective wellbeing): the first model focused on effective functioning and the second on subjective capability evaluation. All four models were weighted and adjusted for: gender, age, marital status, labor-force status, recent life events (positive and negative), depressive symptoms, and personality type. Income and education were included as additional controls in the models focusing on evaluation.

Standard controls for sex, age, income, schooling, civil status, employment, status, personality traits, depressive symptoms, and recent negative and positive events were also included. Income and schooling were not considered together with functionings, as they are an indicator of the functionings “basic needs” and “understanding the world” (see Table 1).

All independent continuous variables were centered to the mean to interpret the constant in regression estimates as the adjusted average of the respective subjective wellbeing being explained. Given a high number of missing cases, multiple imputations through chained equations were performed.

As regards the hypotheses formulated in the introduction, multiple linear regression allows validating H1, H2, and H3, identifying the capabilities correlated both with individual and social subjective wellbeing as those statistically significant in the regressions, and H4, through the size and significance of the coefficients.

Aside multiple linear regression, other statistical analyses were performed, such as Generalized Least Squares and Maximum Likelihood, but yielded no significant change in parameter estimates. All these results are strictly correlational and should be interpreted with caution. For this reason, quasi-experimental methods were also applied. The relationship between capabilities and subjective wellbeing was contrasted using a generalized propensity-score matching model (Hirano and Imbens, 2004). The objective of this analysis was to study more precisely the effect of capabilities (in their dimensions of functioning and subjective evaluation) on life satisfaction and social subjective wellbeing. In particular, dose-response matching allows more robust conclusions and extract information for the distribution of capabilities, extending our conclusions beyond average correlations highlighted by multiple linear regression.

Matching methods were originally proposed to estimate the effects of social programs on the participating population, taking into account that the potential impact of a certain program may be different for participants and non-participants. Matching consists of finding, for each of the program participants, one (or more) “clones,” or people who are equivalent to a certain participant in all the observable characteristics except for the fact that they have not been “treated” by the program. Assuming all the relevant differences between people (before the program) are captured in these observable variables—what happens when the result is independent of the treatment allocation given the pretreatment variables (conditional independence assumption)—then the matching method can produce an unbiased estimator of the average impact of the treatment.

There are several statistical methods to match treated and untreated individuals. Propensity score matching, one of the most widely used, employs a predicted probability of group membership (treated vs. untreated group) based on observed predictors. This predicted probability, denominated the propensity score, is usually obtained from logistic regression for each person included in the analysis and similarity in its value is what is used to generate the matches that will be subsequently compared (Rosenbaum and Rubin, 1983).

Much of the work on propensity score analyses have focused on cases where the treatment is binary that is, there are only two groups: treatment and control. But in many observational studies, the treatment may be categorical or continuous. In such a case, one may be interested in estimating the dose–response function where the treatment might take many values.

In the case of the analyzes carried out in this research, individuals receive different intensities of treatment, since the capabilities of each are measured on a continuous scale that goes from 0 to 1, where 0 indicates complete absence of capability and 1 indicates its maximum endowment. This makes the analysis more complex, since a generalized propensity score must be estimated that allows calculating the propensities of each individual to be located at the different levels of treatment, in this case, the different levels of endowment of capabilities.

Hirano and Imbens (2004) developed an extension to the propensity score method in a setting with a continuous treatment. They defined a generalization of the propensity score for the binary case developed by Rosenbaum and Rubin (1983) and denominated it the generalized propensity score (GPS). The GPS has many of the attractive properties of the binary-treatment propensity score. Hirano and Imbens (2004) method was assessed by Bia and Mattei (2008), who provided a set of Stata programs that estimate the GPS. Below, the dose response STATA package provided by these authors is used to estimate the effect of continuous variation of each capability on the outcome of interest—first life satisfaction, then social wellbeing—except when the normality of continuous treatment was not achieved, in which case its glmdose extension developed by Guardabascio and Ventura (2014) is used.

In estimating the propensity scores, the capability whose effect on subjective wellbeing is being evaluated operates as a dependent variable. The GPS were estimated including the following controls: age, marital status, positive and negative events that occurred during the year.

After estimating the generalized propensity scores, regression equations of the following type were calculated:

Equation to estimate life satisfaction:

$$\mathrm{ls}=\theta +\alpha \,p+\beta \,p^{2}+{\mathrm{rp}}^{3}+\gamma \,c+\delta \,c^{2}+{\mathrm{fc}}^{3}+\pi \,\mathrm{cp}+\varepsilon$$

Equation to estimate social subjective wellbeing:

$$\mathrm{ssw}=\theta +\alpha p+\beta p^{2}+{\mathrm{rp}}^{3}+\gamma c+\delta c^{2}++{\mathrm{fc}}^{3}+\pi \mathrm{cp}+\varepsilon$$

Where ls = life satisfaction; ssw = social subjective wellbeing; p = propensity score, c = capability; ε = residual term; θ = constant; α, β, γ, r, δ, f and π = regression coefficients of the respective variables.

Then the expected levels of life satisfaction E (ls/c) and social subjective wellbeing E (ssw/c) are estimated for the different levels of endowment of capabilities or functionings.

Propensity-score matching is helpful to confirm the correlation of certain capabilities with both concepts of subjective wellbeing (H1–H4) and is essential to address H5 and characterize the complex and non-linear relationships between capabilities and subjective wellbeing.

## Results

Table 3 presents the adjusted association between capabilities (measured first as functioning then as evaluation) and the two subjective wellbeing outcomes (first individual or life satisfaction, then the composite index of social wellbeing). The coefficients were standardized to compare the magnitude of the effects between capabilities with different measurement scales.

**TABLE 3:** OLS regression results for the association between capabilities and subjective wellbeing.

	**Individual**		**Social**	
	**Functioning**	**Evaluation**	**Functioning**	**Evaluation**
Health	0.02	0.08**	–0.01	0.03
Needs	0.09***	0.17***	–0.06	0.08**
Self-knowledge	0.00	0.01	0.03	0.03
Understanding	–0.02	–0.03	0.06	–0.01
Pleasure	0.02	0.03	0.08***	0.01
Nature	0.05*	0.02	0.07**	0.03
Participation	–0.01	0.04	0.04	0.05*
Ties	0.11***	0.10***	0.03	0.06**
Respect	0.08**	0.07**	0.21***	0.33***
Project	0.08***	0.05*	0.09***	0.03
Security	0.01	0.02	0.04	0.20***
Male	–0.04	−0.06**	0.03	0.02
Age	0.04	0.03	0.07*	0.09
Divorced	−0.07**	−0.07**	–0.05	−0.06*
Single	–0.01	–0.02	0.01	0.00
Student	0.02	0.01	–0.01	–0.01
Homemaker	0.05*	0.06*	0.06*	0.06*
Retiree	0.01	0.02	–0.01	0.00
Unemployed	−0.05*	−0.05*	0.01	0.03
Negative life events	–0.02	0.00	0.00	0.00
Positive life events	0.05*	0.03	0.03	0.00
Depressive symptoms	−0.24***	−0.19***	0.03	0.05*
Responsible personality	0.00	–0.01	0.00	0.00
Extroverted personality	0.02	0.01	–0.04	−0.05*
Emotional personality	0.06**	0.04	0.02	0.01
Education	–	0.01	–	0.01
Income	–	0.03	–	−0.09***
Constant	6.88***	7.25***	4.90***	4.85***
*R*^2^	0.21	0.26	0.11	0.25
Adjusted *R*^2^	0.21	0.25	0.10	0.24
*N*	2,479	2,479	2,397	2,397

For each subjective wellbeing outcome specified in the top row (individual and social), the table presents two models using alternative measurement approaches to capabilities (functioning and evaluation). Weighted standardized regression coefficients presented. *p < 0.05; **p < 0.01; ***p < 0.001.

Needs, ties, respect, and project have fairly consistent beneficial associations with both subjective wellbeing outcomes. Nature, measured as functioning, is associated with both subjective wellbeing outcomes. Other capabilities have no significant associations with subjective wellbeing outcomes (self-knowledge and understanding), or significant associations were isolated and did not display a clear pattern (health, pleasure, participation, and security). Interestingly, the strong correlation of individual subjective wellbeing with the control depressive symptoms (that might also be considered a proxy for a functioning of mental health) does not extend to social subjective wellbeing.

**FIGURE 1:**
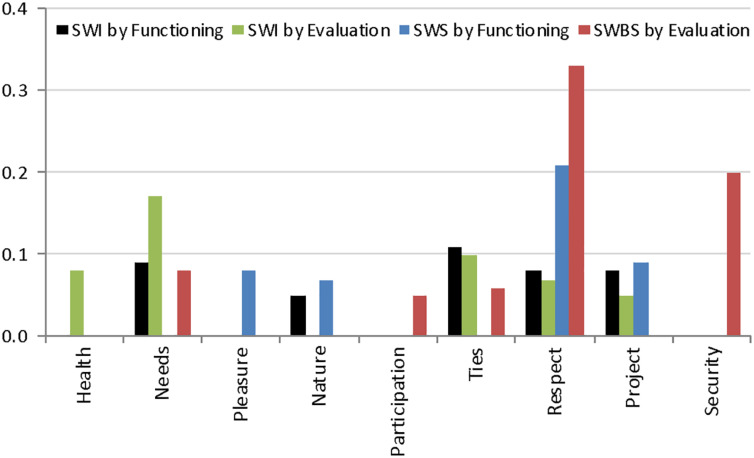
Patterns of systematic associations between capabilities and subjective wellbeing. Bars represent standardized OLS regression coefficients highlighted in yellow in Table 3, all significant at *p* < 05.

Figure 1 focuses on the nine capabilities that had the most consistent and systematic associations with subjective wellbeing outcomes, and presents the standardized regression coefficients highlighted in yellow cells from Table 3. Two clear patterns emerge from this visual representation of the results. First, respect has the strongest and most consistent association with subjective wellbeing, as indicated by the four long bars. Second, capabilities seem to have a slightly stronger association with social subjective wellbeing than with individual subjective wellbeing.

**FIGURE 2:**
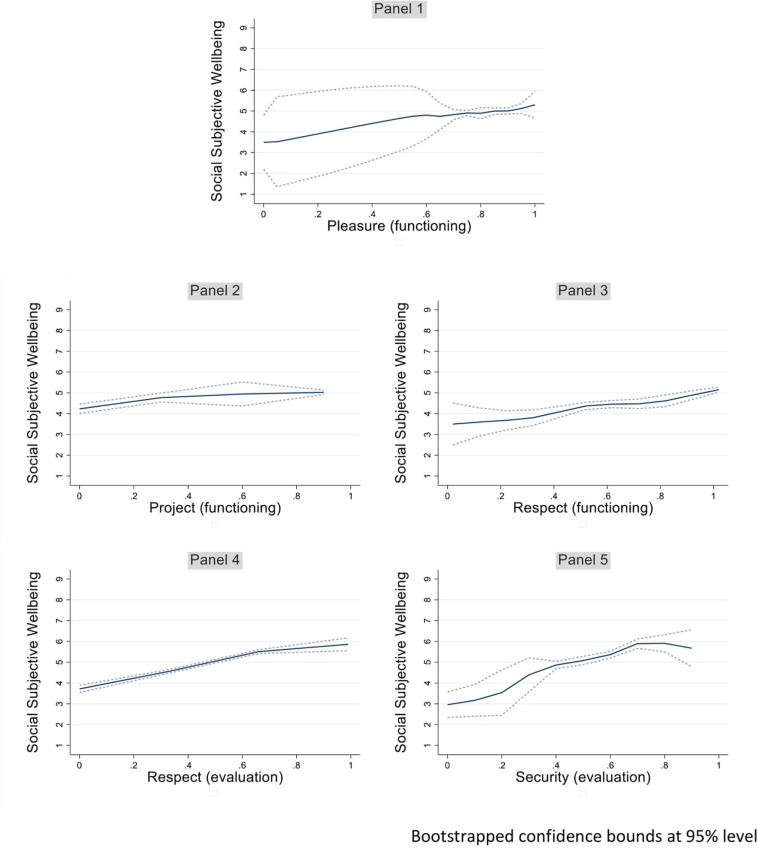
Social subjective wellbeing and capabilities. Bootstrapped confidence bounds at 95% level.

**FIGURE 3:**
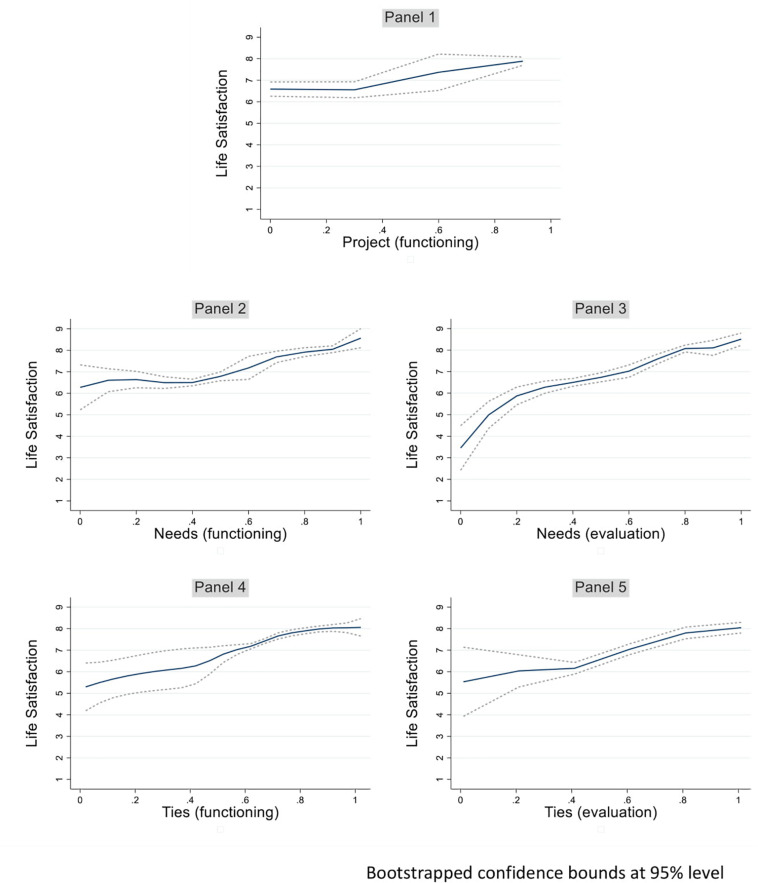
Life satisfaction and capabilities. Bootstrapped confidence bounds at 95% level.

A total of 10 equations were calculated using the methodology of dose response matching to further investigate the most statistically significant relations identified in Table 3 (those with *p* < 0.001). Figure 2 depicts these most significant relations of capabilities and social subjective wellbeing while Figure 3 shows the most significant relations of capabilities and life satisfaction.

In the first place, this analysis confirms the great impact that some capabilities have on both individual and social subjective wellbeing. For example, going from a subjective evaluation of no human security to an intermediate level of the capability almost doubles social subjective wellbeing (see Figure 2, panel 5). Similarly, as shown in panel 3, Figure 3, going from a low subjective evaluation of satisfaction of basic needs to a medium to high perception doubles life satisfaction. This association is replicated quite differently when the analysis involves the actual functioning of the basic needs. In the latter case (Figure 3, panel 2), the increase in life satisfaction is evidenced only when intermediate levels of the capability are achieved.

In the second place, it is interesting to note that the positive impact of an increase in endowment of these capabilities positively impact life satisfaction or social subjective wellbeing in a sometimes non-linear way. For example, panel 1 of Figure 3 describes the effect of the functioning life project as non-existent in the first third of the capability scale, while highly increasing on the second two thirds. Conversely, panel 2 of Figure 2 shows a steeper increase of social subjective wellbeing as the life project capability improves through the first third of the scale, while the curve tends to flatten above that level.

## Discussion

The findings reported underscore the need of a multidimensional perspective on the relationships between capabilities and subjective wellbeing, considering both subjective and objective, as well as individual and social aspects. The results documented consistent associations between dimensions of capabilities exposures (subjective evaluations and effective functionings) and of subjective wellbeing outcomes (individual and social). While there is precedent of the first relationship in terms of correlations between life satisfaction and capabilities, to our knowledge this is the first study to link capabilities with an indicator of social subjective wellbeing integrated by dimensions of trust in institutions and evaluation of social opportunities.

The results for needs, ties, respect, and project are consistent with previous results (Anand et al., 2009; Muffels and Heady, 2013; Graham and Nikolova, 2015; Yeung and Breheny, 2016). Nature measured as functioning is associated with both subjective wellbeing outcomes. This is consistent with the fact that actual contact with nature is the one that makes a difference in individual’s life, not the possibility of having or desire of having more contact. Self-knowledge and understanding the world have no significant associations with subjective wellbeing outcomes, which is also consistent with the literature. In particular, Graham and Nikolova (2015), using quantile regression, are able to discuss differences between groups. These differences appear also when using dose response matching.

The two capabilities that influence the most social and individual subjective wellbeing are “social ties” and “feeling recognized and respected in dignity and rights.” Both capabilities are intimately related with social belongingness. The effect of social ties on individual subjective wellbeing has already been stressed for individual subjective wellbeing and health outcomes such as mortality (see Kawachi and Berkman, 2001; Holt-Lunstad et al., 2010, 2015; Choi et al., 2014; Ehsana et al., 2019). Although not conceptualized as the capability “feeling recognized and respected in dignity and rights,” the experience of discrimination has also been documented to have an impact on individual subjective wellbeing and other health outcomes (Hackett et al., 2019; Jackson et al., 2019; Couto e Cruz et al., 2020), while the presence of social support might mitigate these negative effects of discrimination (Giurgescu et al., 2017). While the relationship with individual subjective wellbeing has been extensively documented in the literature, the relationship of social ties and respect with social wellbeing is a novel contribution of the present paper that deserves further exploration.

These results stress the importance of including measures of subjective evaluations of capabilities and not only functionings as is usually the case in standard surveys.

It is important to acknowledge the limitations of the study. First, it relies on cross-sectional data and therefore it is neither possible to establish causal relationships nor to isolate potential endogeneity or double causality. The information provided is mostly correlational, but nevertheless important to understand the relationships between key variables related to human wellbeing. In addition, using dose response matching allows to disentangle the relationship between subjective wellbeing and different levels of capability evaluation and functioning.

Second, it is not possible to rule out completely confounding variables that might cause omitted variable bias. However, the use of a specially designed survey that included all relevant capabilities as deliberated by different socioeconomic groups and the inclusion of all controls identified by the literature makes the problem less likely than when using existing surveys that have been designed for other purposes.

Third, the estimated relationship holds for the context of Chile at a certain moment in time and is not possible to extent to other societies. However, the sample is representative of this population and therefore contributes to the understanding of the relationship between subjective wellbeing and capabilities evaluation and functioning that should be complemented with other studies.

Nevertheless, these results provide important insights for public policies in societies that are experiencing problems of social belongingness. It is important to stress the size of the effect of respect (feeling recognized and respected in dignity and rights) and human security as determinants of social wellbeing is very large. Not surprisingly, disruptive social movements that Chile experienced during 2011 (the year of the survey) and more recently in 2019, referred to exclusion and discrimination, lack of human security and equalitarian access to social opportunities. If the objective is to reduce social discontent, governments facing similar situations as Chile, might focus on policies that improve “respect” and “human security.”

On the other hand, those concerned about life satisfaction might consider policies removing obstacles to “life projects,” “social ties,” “basic needs,” and facilitating actual contact with nature that also have an effect on social wellbeing although of a lesser magnitude compared to respect and human security. Moreover, respect has an important relationship with life satisfaction but human security appears not to be related. As expected, mental health as functioning, measured by the control depressive symptoms might also be important for life satisfaction.

Furthermore, the non-linear relationship of certain capabilities with subjective wellbeing suggests certain minimum thresholds should be guaranteed, as the effect is large going from complete deprivation to the threshold, while declining afterward. This is the case of human security, project, respect and to a lesser extent, pleasure (experience of pleasurable activities). This suggests the need for universal policies that guarantee access to these capabilities.

Capabilities and subjective states related to social belongingness seem to play a crucial role. Social ties and respect are capabilities that should be of particular concern for public policies as they not only affect both individual as well as social wellbeing but also, as capabilities, are themselves ends of public policies. The article also recognizes that social wellbeing is a subjective state that is important independently of life satisfaction and crucially determined by other variables, and therefore individual and social wellbeing might move in different directions.

Overall, these findings represent challenges for public policy, which require further research on the cultural and structural determinants of these capabilities, including the realm of subjective experience.

Does it mean that the other capabilities are not important? On the contrary, capabilities are ends, as might be subjective wellbeing outcomes. The fact that understanding the world or self-knowledge are not correlated with the assessment that individuals make about their life and their experience of society does not imply that they should not be facilitated by public policies.

Findings from this study have practical and policy implications, and may inform public deliberation processes and policy decisions to develop capabilities, promote subjective wellbeing, and ultimately promote positive belongingness. Future challenges aside measurement and analysis of the determinants of wellbeing, include the necessity to better understand policies that improve capabilities, by their own sake but also as determinants of individual and social wellbeing and social belongingness. Public policies and programs should be evaluated not only on the basis of standard tools such as cost-benefit analysis but also multi-criteria methods that might integrate its effects on other less conventional measures such as the ones considered in this paper.

## Conclusion

In this study we sought to contribute to a multidimensional perspective on belongingness by analyzing the relationship between capabilities (subjective evaluation and effective functioning) with subjective wellbeing (social and individual). We identified (satisfaction of) basic needs, social ties, respect (“Feeling recognized and respected in dignity and rights”), and project (“Having and deploying a life project”) as capability evaluation and functioning that have fairly consistent beneficial associations with both individual and social subjective wellbeing outcomes.

Other statistically significant capability evaluations include “Health” for individual subjective wellbeing and “human security” and “participation and influence in society” for social wellbeing. The variables more correlated with individual subjective wellbeing are basic needs, social ties and respect, both as capability evaluation as functioning. Those more associated with social subjective wellbeing are respect and human security, both measured as functionings.

Capabilities relate to subjective wellbeing outcomes in a non-linear manner, so that the magnitude of the effect usually depends on the initial level of the capability. Inspecting associations only between means of the variables involved, therefore, gives a hint on the existence of a relation but, to further grasp its exact nature, an analysis that takes into account complete distributions is required. This document develops one of the methods that are used to understand distributional effects, dose response matching. Its application not only corroborates that when an increase in the capability endowment has an effect on wellbeing, its magnitude usually depends on the initial level of the capability but also that the relationship is not linear.

Further, a threshold level of certain functionings appears to be necessary for them to have a positive effect on subjective wellbeing. This is the case of project and needs for life satisfaction and, to some degree, respect and human security for social wellbeing. On the contrary, needs as evaluation has a large effect when increasing from very low levels of the capability, while high levels of human security do not longer continue to increase social wellbeing. Social ties have a more continuous positive effect on life satisfaction, both as capability evaluation as well as functioning. Note that this concept is also a measure of experienced links with others and therefore is closely related with social belongingness.

## Data Availability Statement

Publicly available datasets were analyzed in this study. This data can be found here: https://www.estudiospnud.cl/bases-de-datos/encuestas-de-desarrollo-humano-2011/.

## Author Contributions

PG conceptualized the study and led the writing of the manuscript. FD and EC conducted the analyses and contributed to writing the manuscript. All authors contributed to the article and approved the submitted version.

## Conflict of Interest

The authors declare that the research was conducted in the absence of any commercial or financial relationships that could be construed as a potential conflict of interest.

## Footnotes

1 Examples of its influence are Stiglitz et al. (2009) on the measurement of economic performance and social progress, Alkire and Foster (2011) on multidimensional poverty, Sehnbruch et al. (2020) on quality of employment, UNDP’s human development reports that have applied this perspective to the analysis of most social problems or the Gross National Happiness Index that is used instead of GDP growth for project evaluation in Bhutan.

2 Binder (2014) also argued that this would offer a more detailed picture even when subscribing to a welfare approach; the discrepancy in functioning achievement between persons who report the same level of happiness can be understood as a measure of adaptive preferences; policy makers might focus on promoting individual agency and creating an institutional environment that allows individuals to successfully pursue their own conceptions of wellbeing, thereby not reducing them to mere “metric stations” whose happiness rating has to be mechanically increased; and by removing focus on actual measurements of subjective wellbeing it reduces the risk of manipulation (by policy-makers and by citizens) inherent in subjective wellbeing measures.

3 At present, how easy or difficult do you find it to enjoy the love care and support of your immediate family? 28. I have a clear plan of how I would like my life to be. 30. Outside of work, have you recently felt that you were playing a useful part in things?

4 Is your current accommodation adequate or inadequate for your current needs?

5 Do you find it easy or difficult to express feelings of love, grief, longing, gratitude and anger compared to most people of your age?

6 I have already reached a lot of my goals, given my age; I consider myself in good health; I consider myself as well educated; My (household) income is sufficient to live well; I have a good social life (friends, associations, …); I live and spent my time in pleasant environments (house, work, environment); I act according to my personal vision on life when making decisions; I am satisfied with my actual situation (work/study/retirement); I am satisfied with my relationship.

7 “People’s skills, such as ‘being able to imagine the situation of others,’ ‘being able to have a concept of a good life based on one’s own judgment. (say, life-autonomy),’ and ‘self-worth,’ significantly predict well- being, measured as life satisfaction.”

8 “Life satisfaction, is positively associated with some functionings in the following years, such as ‘being healthy’ and ‘having satisfying social relations.’ Also ‘having satisfying social relations’ appears to be positively associated with wellbeing in the following years, so that social relations and wellbeing may have two-way causation.”
